# Commissioning and clinical implementation of an independent dose calculation system for scanned proton beams

**DOI:** 10.1002/acm2.14328

**Published:** 2024-03-29

**Authors:** Ralf Dreindl, Marta Bolsa‐Ferruz, Rosa Fayos‐Sola, Fatima Padilla Cabal, Lukas Scheuchenpflug, Alessio Elia, Antonio Amico, Antonio Carlino, Markus Stock, Loïc Grevillot

**Affiliations:** ^1^ MedAustron Ion Therapy Center Wiener Neustadt Austria; ^2^ Department of Medical Physics and Radiation Protection Hospital Universitario La Princesa Madrid Spain; ^3^ Division Medical Radiation Physics Department of Radiation Oncology Medical University of Vienna/AKH Wien Vienna Austria; ^4^ Department of Isotope Physics Faculty of Physics University of Vienna Vienna Austria; ^5^ Medical Physics Department Veneto Institute of Oncology IOV ‐ IRCCS Padua Italy

**Keywords:** beam time reduction, clinical implementation, commissioning, independent dose calculation, pencil beam scanning, proton therapy

## Abstract

**Purpose:**

Experimental patient‐specific QA (PSQA) is a time and resource‐intensive process, with a poor sensitivity in detecting errors. Radiation therapy facilities aim to substitute it by means of independent dose calculation (IDC) in combination with a comprehensive beam delivery QA program. This paper reports on the commissioning of the IDC software tool myQA iON (IBA Dosimetry) for proton therapy and its clinical implementation at the MedAustron Ion Therapy Center.

**Methods:**

The IDC commissioning work included the validation of the beam model, the implementation and validation of clinical CT protocols, and the evaluation of patient treatment data. Dose difference maps, gamma index distributions, and pass rates (GPR) have been reviewed. The performance of the IDC tool has been assessed and clinical workflows, simulation settings, and GPR tolerances have been defined.

**Results:**

Beam model validation showed agreement of ranges within ± 0.2 mm, Bragg‐Peak widths within ± 0.1 mm, and spot sizes at various air gaps within ± 5% compared to physical measurements. Simulated dose in 2D reference fields deviated by ‐0.3% ± 0.5%, while 3D dose distributions differed by 1.8% on average to measurements. Validation of the CT calibration resulted in systematic differences of 2.0% between IDC and experimental data for tissue like samples. GPRs of 99.4 ± 0.6% were found for head, head and neck, and pediatric CT protocols on a 2%/2 mm gamma criterion. GPRs for the adult abdomen protocol were at 98.9% on average with 3%/3 mm. Root causes of GPR outliers, for example, implants were identified and evaluated.

**Conclusion:**

IDC has been successfully commissioned and integrated into the MedAustron clinical workflow for protons in 2021. IDC has been stepwise and safely substituting experimental PSQA since February 2021. The initial reduction of proton experimental PSQA was about 25% and reached up to 90% after 1 year.

## 1 INTRODUCTION

The MedAustron Ion Therapy Center is located in Wiener Neustadt, Austria, and offers proton and carbon ion treatments. It is one out of six dual particle therapy facilities worldwide. Details about the technology available at MedAustron were presented elsewhere,^1^ however basic information for scanned proton treatments available at MedAustron is provided in Section 2.1. For each patient treatment, a Patient‐specific Quality Assurance (PSQA) process must be conducted. PSQA can either be performed via measurements (experimental PSQA), or using an Independent Dose Calculation (IDC) system, considered as virtual PSQA. Alternative hybrid methods using beam delivery log‐files have also been proposed and can be seen as partly experimental and partly virtual. They require the delivery of treatment fields, but can also be used in combination with an IDC system. Experimental PSQA includes the beam delivery workflow. However, the phantom geometry used to perform the measurements is usually a homogeneous phantom, placed at a fixed position in the treatment room. It is neither representative of the patient anatomy, nor the treatment position. In contrast, IDC allows the recalculation of the treatment plan in the full 3D patient geometry. It considers all Treatment Planning System (TPS) DICOM treatment information. Further, IDC is only virtual and thus does not necessitate any beam time.

There is evidence that IDC, even including simple secondary dose check for 3D conformal radiation therapy, is more sensitive and reliable in catching errors than experimental PSQA. According to ICRP publication 112,^2^ about the prevention of accidental exposure from new external beam radiation therapy technology, the “Calculation of the number of MUs for each patient independently from the TPS would have avoided most of the major accidental exposures resulting from the misuse of a TPS.” (MU stands for Monitor Unit). For IMRT, IDC was shown to be 12 times more sensitive to catching errors than standard experimental QA^3^ and the following conclusion was provided: “A simple recalculation outperformed current measurement‐based IMRT QA methods at detecting unacceptable plans. These findings highlight the value of an independent recalculation, and raise further questions about the current standard of measurement‐based IMRT QA”^3^. Similar reasoning could be applied to light ion beam therapy. Detailed 3D experimental verification of each treatment field is possible, but cannot be achieved in clinical routine, as it would require an enormous amount of beam time. Therefore, experimental PSQA is usually limited to point dose verifications for a few measurement depths per beam. Taking this into consideration and knowing the specificities of scanned proton beam delivery, any beam delivery failure transversal or proximal to the detector position may be completely missed. Consequently and like for IMRT, the question about the current standard of measurement‐based PSQA should be raised for proton therapy as well.

Several groups worked on the implementation of proton IDC systems in their clinics and developments were often tailored to facility specificities.^4^, ^5^, ^6^, ^7^, ^8^, ^9^, ^10^, ^11^, ^12^ Using a Monte Carlo (MC) algorithm in an IDC system was shown to pinpoint dose computation issues from analytical proton algorithms implemented in the TPS. These errors would not have been detected using experimental PSQA.^13^ Further, setting up IDC processes in a clinic can save lots of beam time that would be blocked by experimental PSQA otherwise. In another study, log‐file based IDC was shown to be more effective in detecting data transfer failures than experimental PSQA.^14^ IDC was able to detect 11 out of 21 failure scenarios, while experimental PSQA was only able to detect 1 out of 21 failures. This highlights once again the poor effectiveness of the experimental PSQA process at detecting treatment errors. We would like to emphasize that IDC is a QA tool that should be used complementary to QA measurements. The question is rather to know whether beam time is better invested into PSQA measurements or more specific beam delivery QA measurements complemented by patient specific IDC. Based on experimental PSQA experience of more than 2700 plans, the internal analysis performed at our facility (out of scope for this paper) led us the second option. Experimental PSQA was substituted step‐wise by IDC over a period of about 1 year and ramped‐up to 90% of the proton cases. Together with the implementation of IDC, our beam delivery QA program was complemented by a small set of PSQA‐like constancy tests on a monthly basis.

This paper presents the commissioning and clinical implementation of myQA iON (IBA Dosimetry, Schwarzenbruck, Germany). myQA iON is a commercial CE‐marked product, relying on the MCsquare dose engine.^15^ It allows IDC for scanned proton beam delivery. At first, MedAustron proton treatment specificities are provided (Section 2.1) and a description of myQA iON (Section 2.2) is given. Subsequently, the IDC commissioning concept is presented in Section 2.3. It holds the methodology about the beam model commissioning (Section 2.3.1), CT calibration validation (Section 2.3.2), and clinical commissioning (Section 2.3.3). The result section includes the beam model validation in 1D, 2D, and 3D (Section 3.1), and CT calibration results in an anthropomorphic phantom (Section 3.2). A detailed evaluation of the clinical commissioning based on patient treatment data was used to define IDC tolerances and fail levels (Section 3.3). Finally, the discussion of the key results yields to the conclusion of the work. The methodology used for the clinical implementation is reported in detail and could serve other clinics. To our best knowledge, this is the first time a light ion beam therapy center reports the substitution of experimental PSQA by up to 90% using an IDC system.

## 2 METHODS

### 2.1 MedAustron proton beam delivery specificities

#### 2.1.1 MedAustron therapy accelerator and dose delivery system

The MedAustron Particle Therapy Accelerator (MAPTA) was developed within a collaboration between CERN, CNAO, and MedAustron.^16^ As one of its main components, MAPTA holds a synchrotron which is accelerating protons and carbon ions to their prescribed energies. For proton MAPTA provides 255 clinical energies ranging from 62.4 to 252.7 MeV. Clinically, these energies translate to beam ranges (R_80_) in water between 30 and 380 mm, where R_80_ is defined at the 80% dose level in the distal fall off of the Bragg‐Peak. After reaching its desired beam energy, the particle beam is extracted from the synchrotron and guided through the high energy beam transfer line up to the treatment nozzle of one of three clinical treatment/irradiation rooms (IR2, IR3, and IR4) or one research irradiation room (IR1). IR2 holds one horizontal and one vertical beam line (HBL, VBL) for proton and carbon ions. IR3 provides a horizontal proton beam line. IR4 is a proton rotating gantry room featuring delivery angles between 0° and 180°.

Inside the treatment nozzle of every beam line, the dose delivery system is controlling and monitoring the particle beam with respect to its lateral dimensions (full width half maximum FWHM), to its lateral positions perpendicular to the beam direction, and to its delivered number of particles (NP). A range shifter (RS) of 35 mm water equivalent thickness (WET) can be inserted into the beam path to deliver dose to superficial depths.

#### 2.1.2 Non‐isocentric proton treatment delivery

Proton beam scattering has a large impact on the treatment penumbra. Strategies to reduce the treatment penumbra foresee the reduction of materials inside the nozzle to an absolute minimum and/or to minimize the distance between nozzle window and patient surface. The latter can be achieved by using extendible snouts or by using non‐isocentric treatment setups, where the patient is moved as close as possible towards the nozzle window.^17^ At MedAustron, non‐isocentric setups are used for clinical treatments in the fixed beam lines (IR2 and IR3), while the patient is positioned with a seven‐degree of freedom ceiling‐mounted positioning system.^1^ Leaving the idea of an isocentric setup behind, beam delivery commissioning certainly has to investigate many non‐isocentric scenarios to assure safe and high‐quality treatments delivered to the patients. Hence, the commissioning is not only done with detectors set up at isocenter, but also at distances upstream toward the nozzle. The concept of Isocenter to Surface of detector Distances of x cm (ISDx), where ISD50 means a 50 cm distance from the isocenter toward the nozzle, was therefore defined. The concept was used throughout the beam delivery and TPS commissioning work at MedAustron.^18^, ^19^ ISD50 was chosen as a representative position for the evaluation of non‐isocentric irradiation. This point is called Non‐Isocentric Reference Point (NIRP).^17^ The nominal distance between the isocenter and the nozzle exit window in all HBL and VBL beamlines is specified to be 65 cm.

### 2.2 myQA iON v1.2.0

myQA iON is a server‐based software used to perform PSQA for proton therapy. It is based on several, partly open‐source, research tools^20^ and allows the user to verify a patient treatment plan by using the independent Monte Carlo dose computation algorithm MCsquare.^15^ It computes a dose distribution based on a clinically approved RT Ion plan, CT and RT structure set. The scoring resolution of the myQA iON Monte Carlo simulation in version v1.2.0 is equivalent to the resolution of the input DICOM CT dataset. myQA iON also provides a 3D gamma index analysis comparing the TPS dose and the dose resulting from the independent Monte Carlo simulation. The gamma index calculation in myQA iON has been implemented according to Chen et al.^21^ myQA iON v1.2.0 was installed on a Dell Power Edge R630 Server with an Intel Xeon CPU E5‐2697 holding 24 cores. At the time of installation, 32 GB RAM and 500 GB HDD redundant data storage were assigned. The data storage will be increased on demand.

CT Hounsfield units (HU) have to be transferred into physical quantities required for the Monte Carlo simulation. In order to do so, myQA iON uses CT calibration curves (provided by the user) to convert HUs of all voxels of the patient CT into mass densities and compositions as defined by Schneider et al.^22^

The Monte Carlo simulation settings allow the user to define the minimum number of primaries per simulation, as well as a maximum statistical uncertainty of the resulting simulation. The production of nuclear products can be deactivated completely or selectively for secondary protons, deuterons, and alphas. The energy cut below which nuclear charged secondaries are locally absorbed $Ecut_{pro}$, the maximum distance between two steps $D_{max}$, the maximum fractional energy loss per step $\varepsilon_{max},$ and the energy threshold for the production of secondary electrons $Te_{min}$ can be defined.^15^ All these parameters can be adjusted to find a trade‐off between simulation time and accuracy. The settings listed in Table 1 were used throughout the entire commissioning. Simulated primaries and uncertainty were depending on the performed test and are listed in the respective sections.

**TABLE 1:** MCsquare simulation settings used during the commissioning work.

MCsquare simulation setting	
Energy cut $Ecut_{pro}$	0.5 MeV
Maximum distance $D_{max}$	0.2 cm
Maximum fractional energy loss per step $\varepsilon_{max}$	0.25
Minimum energy transfer $Te_{min}$	0.02 MeV
Nuclear interactions	On
Secondary protons	On
Secondary deuterons	On
Secondary alphas	On

### 2.3 IDC commissioning concept and workflows

The commissioning of myQA iON IDC was divided into three major parts and consisted of (1) the dosimetric commissioning of the beam model, (2) the CT calibration, and (3) the clinical commissioning. An overview of all tests carried out in this work is given in Table 2. It lists the tested energies, specifies the used ISDs, and summarizes the myQA iON simulation parameters. As a general concept throughout this work, representative energies as listed in Table 3 have been selected from the full range of the clinical proton beam energies and were repeatedly used: 5 key ∈ 9 calibration ∈ 20 Major ∈ 255 clinical energies. On top, four RS verification energies had been defined. The dosimetric commissioning is always performed by comparing myQA iON against reference beam delivery commissioning measurements.^18^

**TABLE 2:** Overview of the tasks performed throughout the commissioning of myQA iON.

Commissioning task	Test description	Beam energy	ISD	Simulation settings
**A. Beam model commissioning**				
1D delivery	R_80_ and BPW_80_ in water	20 Major 5 key	ISD0 ISD50	1E7 primaries
	R_80_ and BPW_80_ with RS in water	4 RS verif	ISD50	1E7 primaries
2D delivery	Spot size in air	20 Major	ISD0, ISD40, ISD50, ISD58	1E7 primaries
	Spot size in air	5 key	ISD10, ISD20, ISD30	1E7 primaries
	Spot size with RS in air	4 RS verif	ISD0, ISD50	1E7 primaries
	VSAD	min, medium, max	ISD0, ISD40, ISD50, ISD58	1E7 primaries
	Dose in reference conditions: 12 × 12 cm^2^ field with 2 mm spot spacing at 14 and 20 mm depth with ROOS ionization chamber (PTW‐34001)	20 Major	ISD0	1E7 primaries
3D delivery	3D cubic reference boxes w/wo RS	Modulated scanned beam	ISD0, ISD50	1E8 primaries; 1.5% uncertainty
	Regular shaped fields w/wo RS in water	Modulated scanned beam	ISD0, ISD50	1E8 primaries; 1.5% uncertainty
	Irregular shaped fields w/wo RS	Modulated scanned beam	ISD0, ISD50	1E8 primaries; 1.5% uncertainty
	Regular shaped fields with RS at different air gaps	Modulated scanned beam	ISD0, ISD10, ISD20, ISD30, ISD40, ISD50	1E8 primaries; 1.5% uncertainty
**B. CT calibration validation**				
Validation of rWET	rWET of CIRS tissue equivalent slabs (1 and 2 cm)	160.0 MeV, 198.0 MeV	ISD0	5E7 primaries; 1.5% uncertainty
**C. Clinical commissioning**				
Benchmarking of gamma index	Comparison to gamma index computed with Verisoft (PTW)	Modulated scanned beam		5E7 primaries; 1.5% uncertainty
Definition of clinical simulation parameters	Testing of various simulation parameters as trade‐off between computation times vs. accuracy	Modulated scanned beam		Variable settings
PSQA workflow	Computation of clinical plans in water and comparison against experimental measurements	Modulated scanned beam		5E7 primaries; 1.5% uncertainty
Clinical workflow	Evaluation of clinical plans in myQA iON	Modulated scanned beam		5E7 primaries; 1.5% uncertainty
Assessment of gamma criteria	Definition of IDC tolerances and fail levels	Modulated scanned beam		5E7 primaries; 1.5% uncertainty

BPW_80_, Bragg Peak width at the 80% dose level; rWET, relative water equivalent thickness; VSAD, virtual source axis distance; w/wo, with/without.

**TABLE 3:** List of proton beam energies repeatedly used throughout the commissioning work.

Energy commissioning classification	Beam energies (MeV)
Key energy	62.4, 97.4, 148.2, 198.0, 252.7
Calibration energy	62.4, 81.3, 97.4, 124.7, 148.2, 179.2, 198.0, 224.2, 252.7
Major energy	62.4, 72.4, 81.3, 97.4, 111.6, 124.7, 136.8, 148.2, 159.0, 169.3, 179.2, 188.7, 198.0, 207.0, 215.7, 224.2, 232.6, 240.8, 248.8, 252.7
RS verification energy	97.4, 124.7, 148.2, 198.0

#### 2.3.1 Beam model commissioning

The dosimetric beam model commissioning was divided with respect to delivery complexity into 1D, 2D, and 3D. The 1D delivery involves the delivery of a single static pencil beam. The 2D delivery is the delivery of a quasi‐discrete scanned pencil beam for a single energy layer. The 3D delivery is the delivery of quasi‐discrete scanned pencil beam for multiple energy layers to accomplish a 3D dose distribution inside a target. This concept was previously used and described elsewhere with respect to the dosimetric beam line commissioning^18^ and the commissioning of clinical beam models for protons in the MedAustron TPS RayStation (RaySearch Laboratories, Sweden).^19^

Experimental data for beam modeling has been acquired during the commissioning of the proton HBL.^18^ The reader is further invited to refer to the work of Grevillot et al. for details about the measurement equipment and methodology.^23^, ^24^ Some of the commissioning measurements were used for TPS and myQA iON beam modeling, others were used for validation. myQA iON commissioning set‐ups were prepared in the TPS. The respective treatment plans, planning CTs, and structure sets were exported to myQA iON as inputs to run the simulations. The resulting 3D DICOM dose distributions computed with IDC were exported and beam delivery commissioning parameters for R_80_, Bragg peak width at the 80% dose level (BPW_80_), spot optics (FWHM, position), dose in reference conditions in water, and point doses in Spread Out Bragg‐Peaks (SOBPs) were extracted utilizing the in‐house *Toolkit for the evaluation of 3D DICOM dose Distributions* (*T3DD*).^25^ Commissioning of integrated radial profiles as a function of depth (IRPD) for energies below 150 MeV were influenced by the simulation's scoring resolution in the initial simulation setup (CT slice thickness of 2 mm in beam direction). A workaround for these energies was defined by making use of the lateral CT resolution (0.68 mm) in order to overcome the under sampling of the extracted Bragg‐peak curve (see Discussion section for more details). The correct implementation of the beam model's Virtual Source Axis Distance (VSAD) was verified by evaluation of spot positions at different airgaps between 6.4 cm (ISD58) and 64.8 cm (ISD0).

#### 2.3.2 CT calibration validation

CT Hounsfield units (HU) have to be transferred into physical quantities required for the Monte Carlo simulation. Conceptually, this conversion differs between myQA iON and the RayStation. Both the TPS and the IDC tool are using HU to mass density relations known as CT calibration curves to convert HUs of all voxels of the patient CT into mass densities. In addition, myQA iON utilizes an unchangeable materials‐list based on Schneider‐tables, to define intervals used to convert the voxels HU into the respective material compositions.^22^ In contrast, the TPS facilitates a materials‐list based on data from ICRU report 49,^26^ ICRU report 44,^27^ and ICRU report 23,^28^ to define fractions of atomic elements and mean ionization potentials for every single voxel within the simulated volume. With respect to handling the end of the CT scanner's range, the different material lists lead to following differences: myQA iON assigns all voxels with HU higher than 1500 HU to a dense bone material composition as defined in Schneider et al.,^22^ while the TPS resolves the upper end of the HU range with bone materials (∼500 to 2432 HU), Al (2433 to 2832 HU), and Fe (> 2833 HU).^29^

There are eight different CT protocols and respective HU to mass density curves in use clinically in the MedAustron TPS. Their implementation and validation were presented previously.^30^ At present, for myQA iON six out of these eight CT calibration curves have been implemented and validated in myQA iON. The CT calibration curves correspond to different body locations for adults and pediatric patients as presented in Table 4. Their implementation into myQA iON was done by using the identical CT calibration curves (HU to density) as for the TPS. A total of 13 different CIRS tissue equivalent samples of 1 cm and/or 2 cm physical thickness have been modelled in the TPS and forwarded to the IDC for simulation. Namely, these samples were lung inhale/exhale, adipose, soft tissue breast, water, muscle, brain, prostate, 2 x trabecular bone, 2 x dense bone and cortical bone.

**TABLE 4:** Distribution of treatment plans for clinical workflow testing, classified according to their target location and CT protocol.

			Treatment plan complexity and modulation		
CT protocol	CT slice thickness (mm)	Lateral pixel spacing (mm)	SFO	MFO	Sum
Adult head	2.0	0.68/0.68	35	39	74
Adult H&N	3.0	0.98/0.98	4	3	7
Adult abdomen	3.0	1.17/1.17	15	14	29
Pediatric head	2.0	0.68/0.68	17	1	18
Pediatric abdomen	3.0	0.78/0.78	–	1	1
Infant body	2.0	0.68/0.68	2	–	2
Sum			73	58	131

Abbreviations: MFO, multiple field optimization; SFO, single field optimization.

The methodology intended the measurement of the beam range R_80_ of an open proton beam (i.e., without RS) for beam energies 160.0 and 198.0 MeV, as well as the measurement of the same beam passing through the tissue‐like slabs, mounted in front of a water phantom. For validation, WET values of the experimental data have been compared to simulations of the same setup in myQA iON and in RayStation, thereafter.

The simulated relative WET of the material (rWET_m_) is given as:

$$rWET_{m}=\frac{R_{80,w}-R_{80,m}}{t}$$

with $R_{80,w}$ and $R_{80,m}$ being $R_{80}$ in *water* behind the *material* slab. Further, t is indicating the thickness of the sample.

The difference in rWET in % between simulation (IDC and/or TPS) and measurement for material m is defined as

$$\Delta rWET_{m}\left[\%\right]=\frac{rWET_{m}-rWET_{m,experimental}}{rWET_{m,experimental}}\cdot 100$$

The CT scanner at our institute is of type Philips Brilliance CT Big Bore Oncology (Philips, Netherlands) covering HU ranges between −1024 and 3071 HU.

#### 2.3.3 Clinical commissioning

Clinical commissioning presents the final step before implementing the IDC into the clinic. At first, the implemented gamma index method was benchmarked by comparing myQA iON and RayStation 8B dose distributions of overall 71 beamsets in myQA iON and VeriSoft 7.1 (PTW, Freiburg, Germany). Gamma index distributions and gamma pass rates (GPRs) were evaluated. Both datasets had been normalized to the D_max_ of the TPS dose distribution. Only dose voxels above 10% of the D_max_ were considered. Dose difference and distance to agreement (DTA) of 2%/2 mm and 3%/3 mm have been used to calculate the three‐dimensional gamma indices.^31^, ^32^, ^33^, ^34^

The accuracy of the myQA iON dose computation was thereafter tested for 131 clinical treatment plans of varying complexity and modulation: Single field optimization (SFO), Multiple Field Optimization (MFO). The clinical plans were recalculated on their respective planning CT (*Clinical workflow*) for the six clinical CT protocols. The CT protocols and treatment plans are listed in Table 4. The CT slice thickness varied between 2 mm for adult and pediatric head or infant protocols, and 3 mm for adult head and neck (H&N) or abdomen protocols. The lateral pixel spacing in orthogonal directions is depending on the protocol and ranged between 0.68  and 1.17 mm.

In addition, 79 treatment plans had been recalculated in a water phantom (*PSQA workflow*) and compared to results from measurement based PSQA, where point doses from 24 PinPoint ionization chambers type 31015 (PTW) inside a 3D detector block were used.^35^, ^36^

Default simulation parameters for the *clinical workflow* and the *PSQA workflow* were defined as 5E7 primaries and a minimum uncertainty of 1.5%. These default settings have been fixed upfront, after varying the number of primaries (1E7 to 1E8) and the minimum uncertainty of the Monte Carlo simulation (1%, 1.5%, and 2%) on a selected set of cubic dose distributions and clinical cases. They have been defined as a compromise between statistical uncertainty and computation time. Further, the assessment of reasonable clinical parameters was also proven mathematically: The scoring volume in myQA iON is equivalent to the CT resolution (0.92 mm^3^ considering CT slice thicknesses of 2 mm with a lateral resolution of 0.68 mm). It is about nine times smaller than in the scoring volume used clinically in RayStation (8 mm^3^ considering an isotropic 2 mm scoring grid), where we aim for an uncertainty of 0.5% for clinically approved treatment plan dose distributions. Considering that the statistical uncertainty is invert proportional to the square root of N with N being the number of primaries, we approximated that 0.5% statistical uncertainty in 8 mm^3^ voxels is equivalent to about 1.5% statistical uncertainty in 0.92 mm^3^ voxels. The clinical gamma criteria and GPR were defined as outcome of the statistics from the *clinical workflow* and are presented later.

## 3 RESULTS

### 3.1 Beam model commissioning

#### 3.1.1 1D/2D delivery

Simulated and measured ranges agreed within +0.1 mm and −0.2 mm on R_80_, independent of the simulated setup, for example, without RS at ISD0/ISD50 and with RS at ISD50. The agreement for BPW_80_ was found to be always within ± 0.1 mm.

**FIGURE 1:**
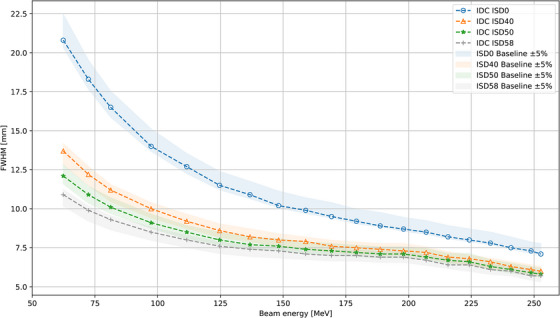
Agreement between simulated and measured FWHM in open beams at various ISDs. Dotted lines indicate IDC simulations. The shaded areas represent ± 5% of the respective baseline.

Investigations regarding 2D optics showed agreement of simulated FWHM against commissioning baseline data within ± 5% for the 20 Major energies at ISD0, ISD40, ISD50, and ISD58 (Figure 1). Inserting the RS into the beam path did not affect the level of agreement with differences of 4.0 ± 0.5% and 3.1 ± 0.9% for ISD0 and ISD50, respectively. The FWHM was found to be within ± 5% for the four RS‐verification energies at ISD0 and ISD50 with a maximum deviation of 4.7%. Open beam setups for additional airgaps at ISD10, ISD20, and ISD30 for the five key energies had been simulated for completeness. The simulated spot sizes at ISD10, ISD20, and ISD30 followed the simulation data for ISD0 and ISD40 and complemented the investigations for 2D spot sizes. Spot positions in orthogonal directions were extracted at four different airgaps (ISD0, ISD40, ISD50, and ISD58) and showed an agreement to the baseline data of −0.3 ± 0.1 mm  and 0.4 ± 0.1 mm, respectively, for horizontally and vertically deflected beams. The systematic differences can be assigned to non‐perfect centering of the beams used in the TPS plans and lateral CT‐resolution limitations. Hence, the VSAD was found to be correctly implemented in the myQA iON beam model.

Simulated doses in reference conditions differed from measured doses on average by −0.3 ± 0.5%. Energies below 100 MeV showed the largest deviations with up to −1.5% for 62.4 MeV. The higher differences are a consequence of the increasing dose gradient (up to 2.7%/mm) and hence larger positioning uncertainties at the measurement and simulation depths for energies below 100 MeV.

#### 3.1.2 3D delivery

Cubic reference and regular boxes at ISD0 deviated by 1.4 ± 0.7% on average. Boxes without RS at ISD50 were off by 1.6 ± 0.8% on average. Boxes at ISD50 with RS showed deviations of 2.3 ± 1.2% on average. Irregular targets at ISD0 and ISD50, with and without RS showed differences of 1.5 ± 0.4% on average. A study on the air gap influence on boxes with RS resulted in deviations of 1.4 ± 0.4% between simulations and measurements on average. The airgap was varied between 14.8 and 64.8 cm.

**FIGURE 2:**
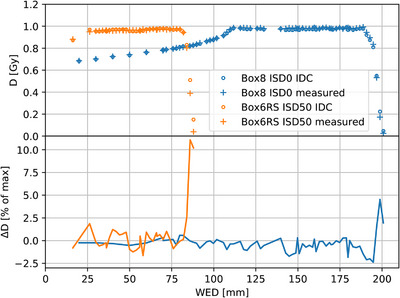
Agreement of IDC simulation and experimental measurements for cubic dose distributions of 8 × 8 × 8 cm^3^ centered at a Water Equivalent Depth (WED) of 15 cm (Box8) and of 6 × 6 × 6 cm^3^ centered at a WED of 5 cm with RS inserted into the beam path (Box6RS).

As a consequence of these findings for 3D dose distributions, the NP per MU were modified and scaled up in the beam model. An averaged scaling factor of −1.8% was applied. Hence, the agreement improved to an overall average of −0.2 ± 0.4% and the beam model was frozen for the consecutive clinical commissioning studies. An example of the agreement of simulated and measured SOBPs after rescaling for one cubic reference box at ISD0 and one at ISD50 with RS can be seen in Figure 2.

### 3.2 CT calibration validation

**FIGURE 3:**
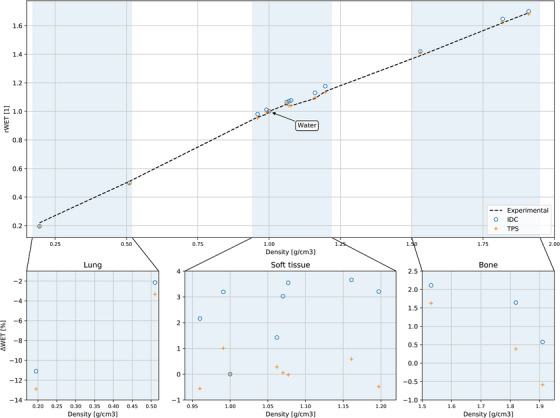
Validation of CT calibration curve (‐) for the adult head protocol. Simulated rWET in myQA iON and TPS as well as ΔWET with respect to experimental measurements of CIRS tissue‐like slabs are shown.

The study on tissue‐like samples was carried out as a comparison between the IDC, the TPS, and experimental WETs. Results are presented in Figure 3. Simulated WETs in myQA iON differed on average by 1.9 ± 1.7% from the measured WETs. The agreement of TPS‐WETs and experimental WETs was −0.1 ± 1.2% and, consequently, myQA iON was underestimating the proton ranges by about 2.0% compared to the TPS and the experimental data. It must be emphasized that tissue‐like samples were overridden by the exact composition and ionization potential provided by the manufacturer for the TPS evaluation. In contrast, this capability being absent in myQA iON, Schneider calibration curves were used as such in myQA iON. Considering the different tissue types, the largest deviations between the WETs simulated in myQA iON and the experimental WETs have been found for lung samples, where deviations for the *lung inhale* tissue‐like sample reached −11.1%. The *soft‐tissue* like samples appeared to have 2.2 ± 1.3% higher WETs, while numbers for dense and cortical bone samples were 1.4 ± 0.8% higher as well.

### 3.3 Clinical commissioning

#### 3.3.1 Clinical computation times

The default parameters listed in Sections 2.2 and 2.3.3 have been used for all simulations carried out during the course of the clinical commissioning. For the installed system, the overall average computation times were 17.4 min, ranging from 5.1 to 94.5 min. Beam sets that were planned on CTs with 2 mm slice thickness finished on average after 10.2 min, while simulations of beamsets on 3 mm CTs required 33.2 min. The simulated treatment site and the target volume relates to the used CT slice thickness as presented in Table 4.

#### 3.3.2 Benchmarking of gamma index

**FIGURE 4:**
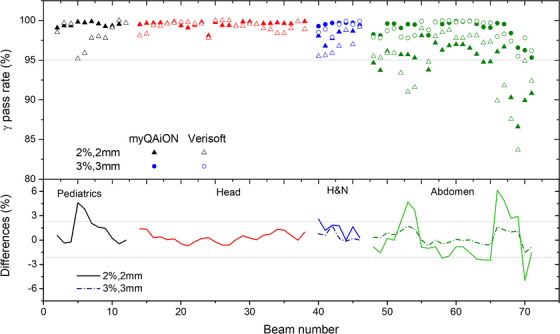
GPRs of 79 beamsets calculated in myQA iON and VeriSoft with 2%/2 mm and 3%/3 mm (H&N and Abdomen protocols).

Results of the gamma index benchmarking are presented in Figure 4. Due to low statistics in pediatrics, results for pediatric head, abdomen, and infant body protocols are grouped together. In contrast, adult protocols are presented separated (Head, H&N, Abdomen). GPRs were found to agree within ± 2.0% on 2%/2 mm for Head and H&N plans between myQA iON and Verisoft. Abdomen plans showed differences of up to 6% in GPR on the 2%/2 mm criterion, while for 3%/3 mm the agreement was found to be within ± 2.0%, as well. For pediatrics plans, the average deviation was found to be 1.3 ± 1.8% on 2%/2 mm and reached up to 4.6% for one treatment plan.

#### 3.3.3 Clinical workflow

**FIGURE 5:**
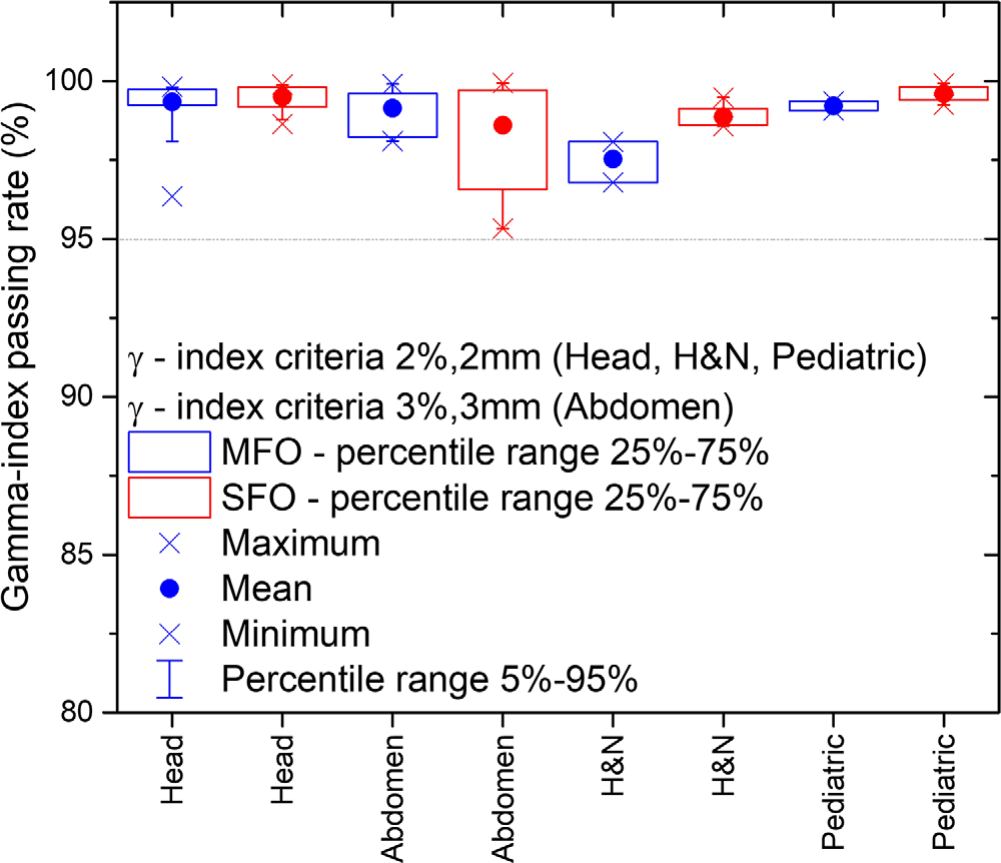
GPR boxplots of 131 clinical beamsets for head, abdomen, H&N, and pediatric CT protocols. The data is presented for SFO and MFO treatment plans. The gamma criteria for head, H&N, and pediatric CT protocols were set to of 2%/2 mm. The gamma criterion of abdomen patients has been increased to 3%/3 mm.

Overall, 131 clinical treatment plans have been simulated in myQA iON and Figure 5 provides statistics of the resulting GPRs. The results were divided into different CT protocols, thus tumor locations. Head, H&N, and pediatric CT protocols showed average GPRs for a 2%/2 mm gamma criterion of 99.4 ± 0.6%. For the adult abdomen protocol, the GPRs statistics for 2%/2 mm were found to be 94.1 ± 3.3% (min GPR: 86.6%, max GPR: 97.0%) for SFO and 93.0 ± 8.5% (min GPR: 70.1%, max GPR: 99.3%) for MFO, respectively. For the 3%/3 mm gamma criterion, the overall mean GPR increased to 98.8 ± 1.3% (min GPR: 95.3%, max GPR: 99.9%).

**FIGURE 6:**
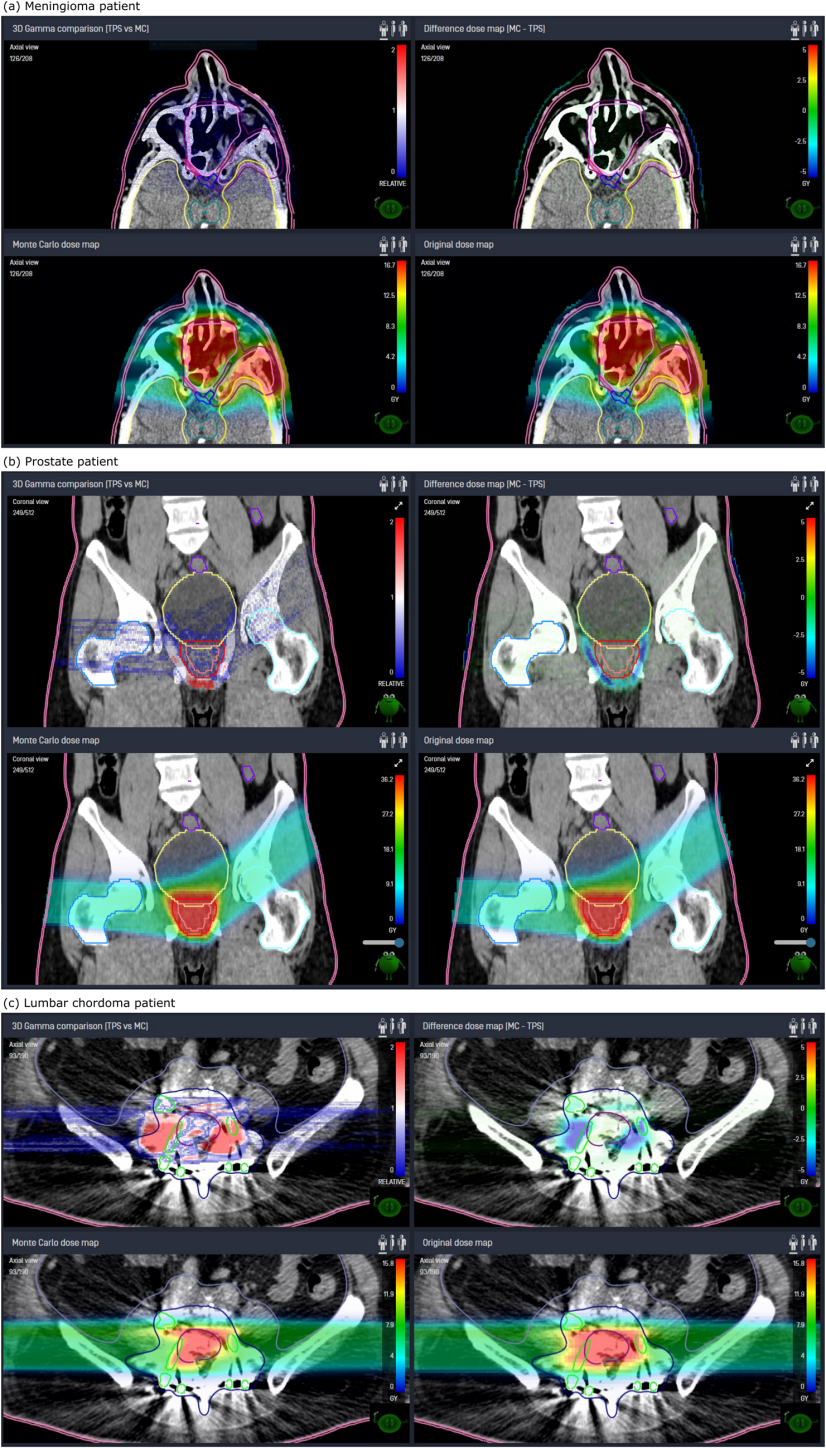
IDC results and clinical examples for (a) a patient suffering from a sinonasal papilloma, (b) a prostate patient, and (c) a lumbar chordoma patient. The four panels present the gamma index distribution, the dose difference map, the TPS dose distribution, and the IDC dose distribution (Starting top left in clockwise order).

Clinical examples are presented in Figure 6, where (a) shows IDC results for a patient suffering from a sinonasal papilloma including infiltration into the left optic nerve and cranial. The resulting GPR of this treatment plan was 99.7%. It indicates very good agreement between the TPS and the IDC dose distribution for the exemplarily sinonasal patient. Figure 6b presents the result of an IDC of a typical prostate cancer patient. GPRs were found to be 94.8% on 2%/2 mm, 96.7% on 3%/2 mm, and 99.2% on 3%/3 mm, respectively, for this patient. In Figure 6c, a patient suffering from a lumbar chordoma in the vertebral spine including resection of LV4 is presented. Implants are causing a partial shielding of the treatment beams. They are indicated with green outline. GPRs were found to be 89.9% on 2%/2 mm, 93.6% on 3%/2 mm, and 96.2% on 3%/3 mm. Further evaluation of the presented cases are available in the Discussion section.

As outcome of GPR statistics and evaluation of patient cases, the clinical gamma criteria were defined with a 2% dose deviation tolerance and a DTA of 2 mm for all CT protocols, except adult abdomen. For the adult abdomen CT protocol, the criterion was defined with 3%/3 mm. Warning and fail levels were defined as 95% and 90%, respectively, for all CT protocols and voxels holding dose values below 10% of the global maximum are ignored from the analysis.

#### 3.3.4 PSQA workflow

The *PSQA workflow* showed an average agreement between point dose measurements and IDC simulations in water of‐0.7 ± 1.2% for more than 1600 point dose comparisons. The agreement of TPS dose distributions to measurements in water have been found to be similar with average deviations of −0.5 ± 1.2%. myQA iON and TPS point dose agreement in water geometry was found to be −0.2 ± 0.5%, ranging from −1.4% to 0.7%.

## 4 DISCUSSION

Results of the beam model commissioning for 1D ranges and 2D optics are presented in Section 3.1. The overall deviations to the baseline data were found to be within clinically acceptable limits (ranges within ± 0.3 mm, spot sizes within ± 5% to baseline). For energies below 150 MeV, the proton Bragg‐Peak is tighter than 4 mm (BPW_80_) in water. As mentioned before, in myQA iON v1.2.0 particle interactions are scored in voxels defined by the CT slice thickness and the lateral resolution of the CT scan. Hence, in situations where the Bragg‐Peak is narrower than twice the scoring voxel dimension, fitting the simulated BP to the measured IRPD is problematic due to under sampling (Nyquist–Shannon sampling theorem^37^, ^38^, ^39^). Rotating the simulation setup and making use of the lateral CT resolution of 0.68 mm solved the under‐sampling issue for 1D and resulted in reliable data for R_80_ and BPW_80_. The comparison of our results to the TPS commissioning results^19^ showed good agreement and further proved that the beam model implemented in myQA iON is meeting the requirements for evaluation and testing in clinical scenarios. A down‐scaling of the NP per MU in the beam model had to be applied based on results from the simulations of 3D dose distributions for targets of different sizes/shapes located at various depths in water with or without RS. The applied −1.8% scaling factor was very similar to the one applied during TPS commissioning previously at our institute (i.e., −2.0% reported in ref. [19]).

The second commissioning step comprised the validation of the clinical CT protocols used at MedAustron. A systematic 2.0% difference between myQA iON and the experimental data as well as to the TPS rWETs was found for tissue‐like samples. The two systems are using different material lists, based on ICRU materials for the TPS and Schneider tables for myQA iON. Uncertainties of tabulated stopping power for elements and compounds are stated to be within 1%−2% and 1%−4%, respectively, according to ICRU report 49.^26^ Hence, the differences in rWET were found to be within stopping power uncertainties. In addition to the validation in plastic, verifications using real tissues, for example, animal experiments are always beneficial. Verification in pig tissues were performed in the past at the time of TPS commissioning at MedAustron. This data will be published in a separate work for protons and carbon ions. WET validation using myQA iON in the pig tissues were performed and agreements were consistent with results extracted from plastic experiments, which were reported in this paper. For instance, agreement for soft tissues was within 2%. The detailed evaluation is out of scope of this paper. However, the differences were accepted and no tuning of the HU to density curves had been applied for the myQA iON CT calibration curves. As a consequence, results of the *clinical workflow* showed notable range differences between myQA iON and TPS. Most dominantly, these differences appear if the treatment beam traverses long distances of soft tissue and bones, as they appear in the pelvis or abdomen. This is a direct consequence of rWET values being systematically higher for myQA iON than for TPS simulations, leading to undershoot of the pencil beams in myQA iON, as compared to the TPS. Practically, the observed 2%‐difference in rWETs convert in a range difference of 2 mm over 10 cm water equivalent distance. Such end of range uncertainties are usually mitigated by beam arrangements from various entrance directions. Typically, at least three beam entrance directions are used in a clinically approved treatment plan at our institute. Handling of high‐density material like bone or implants is difficult for dose calculation algorithms.^40^ In presence of metal implants, HU values saturate and transferred mass densities of the involved voxels are at the maximum of the CT calibration curve. In such situations the used stopping powers for the dose computation are defined by the implemented material lists in the IDC software and the TPS. myQA iON is using a dense Schneider bone, while the TPS RayStation defines highest HU voxels as iron. For the presented beam in the clinical implant example, the used proton energies were between 165.2 and 211.4 MeV. In this energy range, the mass stopping power of Schneider bone is almost 30% higher than the mass stopping power of iron, causing the IDC to stop the proton beam significantly earlier compared to the TPS. The observed shift between the two profiles shown in Figure 8 is consistent with the differences in mass stopping power between the two different materials used for the implant.

The third commissioning step focused on the testing of myQA iON for clinical patients and the evaluation of clinical features (Gamma index, simulation times) provided in the software package. The simulation times depended on many parameters, including the number of simulated primaries, the aimed uncertainty, the target volume, and the treatment depth. It is difficult to identify clear prediction rules, but in general, the most extreme cases appeared for large and deep‐seated treatment volumes, typical for sacral chordomas.

**FIGURE 7:**
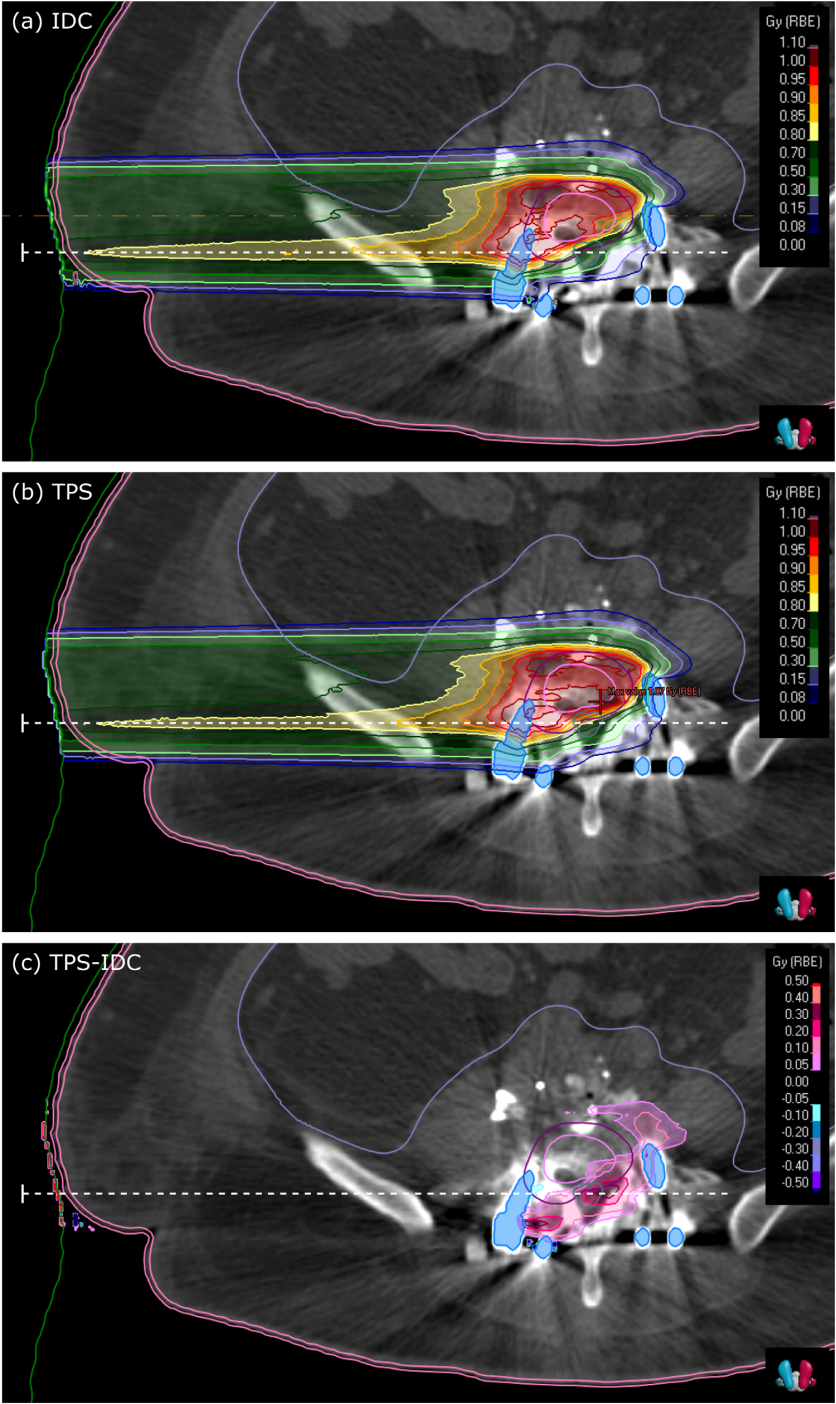
Dose distributions of one of the two opposing beams (right side) shown in Figure 6c for the IDC (a) and the TPS (b). A dose difference plot is shown in (c). The dashed white lines (–) indicate the position of the profiles shown in Figure 8. Names of the delineated structures are listed in Figure 8.

**FIGURE 8:**
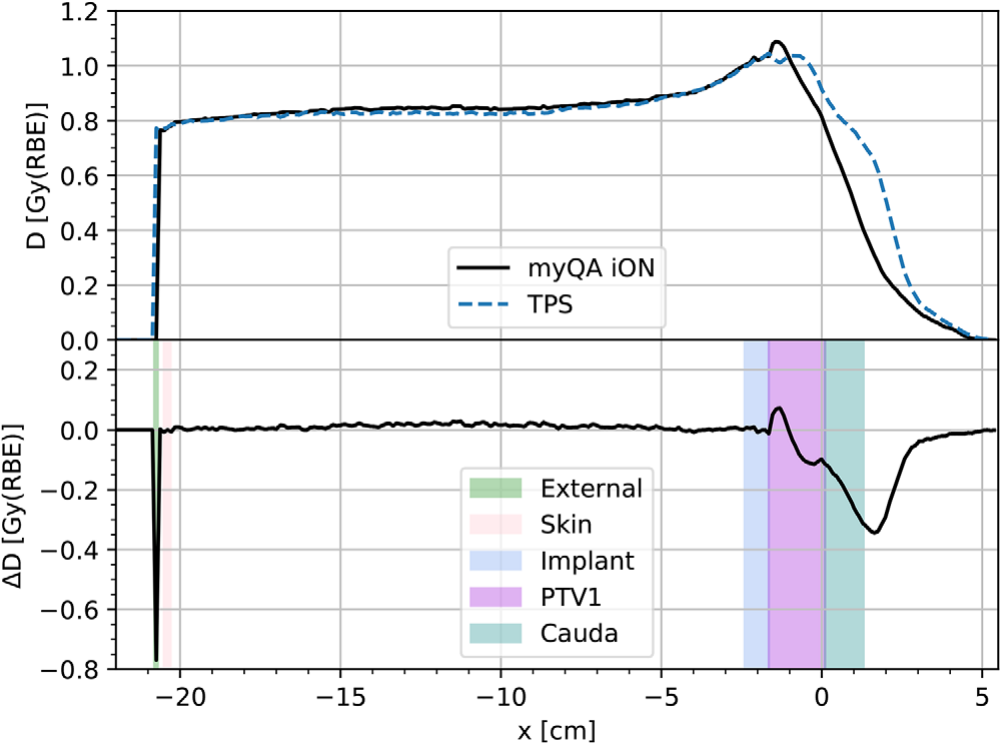
IRPD of the proton beam presented in Figure 7 for a patient with implants in LV4 for myQA iON and the TPS. The structures penetrated by the particle beam in its path through the patient are overlayed in the difference plot.

In general, the crosscheck of gamma indices and pass rates calculated in myQA iON showed that the implemented algorithm is reliable. No significant difference was observed between SFO and MFO plans and only a couple of plans showed GPR below 97.0% using 2%/2 mm. It was possible to assign the root cause for these outliers to one of the following reasons: (1) range uncertainties, (2) field patching inside the target, and/or (3) presence of implants. Typical clinical examples have been presented in Figure 6. For the prostate cancer patient shown in Figure 6b, end of range uncertainties of the two opposed treatment fields (right and oblique‐left) can be seen in the dose difference and the gamma index distribution close to the planning target volume. The chordoma patient Figure 6c is discussed in more detail in the following. The patient underwent vertebral body resection followed by dorsal spondylodesis using Ti‐implants. The implants are causing CT streak artifacts close to the treatment target. Implant CT voxels show HU values of 3071 HU, which is the end of the CT scanner range. The differences between the IDC and the TPS Monte Carlo algorithms in assigning materials to these voxels, leads to differences in deposited doses of up to 36% of the prescription in the close surrounding of the implants for the full beamset. The evaluated beamset consisted of two lateral opposing beams. Details of the beamset's right beam are presented in Figures 7 and 8. The computed dose distributions of myQA iON and the TPS, the dose difference distributions, and an IRPD through the implants are presented. From the dose difference plot and the dose profile, it can be seen that the protons of this beam are reaching deeper by a few millimeters in the TPS compared to IDC. Similar results were found for the opposing beam.

**FIGURE 9:**
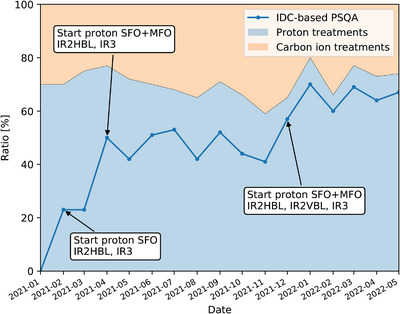
Stepwise increase of IDC and consequently reduction of experimental PSQA for proton HBL and VBL treatment plans since the clinical start of IDC at the MedAustron Ion Therapy Center in February 2021.

After the finalizing the commissioning, the implementation of IDC into the MedAustron treatment workflow was done. A stepwise process was defined to allow a smooth adaption of the clinical workflow without compromising safety for the patients. The transition started with a 2 months’ period in which IDC replaced experimental PSQA only for proton HBL plans with SFO complexity. During this period, all MFO proton HBL plans had still been measured in parallel to the SFO IDC. The rationale of the period was to gain experience with the IDC tool in terms of workflow and knowledge. Further, it aimed to identify limitations of the system for the people involved in the treatment planning and plan review process. Considering all proton and carbon ion treatments for all beamlines available, the first step enabled an overall reduction of experimental PSQA by about 25%. The subsequent inclusion of MFO beamsets further decreased the required beam time and led to an overall reduction of experimental PSQA by 40% to 50% between April and December 2021. In December 2021, the proton VBL was included, and since then, up to 70% of the overall PSQA including carbons and 90% of all proton treatments are done with IDC instead of experimental measurements. The substitution of proton PSQA with IDC resulted in approximatively 250 h of beam time saved in 2021, for a total of about 253 proton patients treated (Figure 9).

IDC already plays an important role in the MedAustron treatment workflow. QA beam time reduction is important to ramp the patient numbers. To achieve this goal, besides improving the performance of MAPTA, IDC for proton treatment plans has a key role. The GATE‐RTion/IDEAL independent dose calculation system for light ion beam therapy will be integrated into myQA iON in the near future and will allow the simulation of carbon ion beam treatments on top of protons.^6^ IDC not only facilitates PSQA, but also eases the treatment adaptation workflow. Of course, treatment safety for our patients has to be guaranteed during this process. Current AAPM recommendation advice to perform experimental QA .^41^ As explained in the Introduction, there is clear evidence of deficiencies of experimental QA to catch errors, as gamma pass rates based on point dose measurements do not provide spatial sensitivity.^2^, ^5^, ^13^ IDC provides simulations on the full treatment volume which are more meaningful and closer to the clinical situation. At the end of the IDC workflow in myQA iON, a single GPR is used to describe the overall outcome of the comparison. On top, a careful review of the dose distribution inside the patient volume is still necessary. For example, a hot spot in the spinal cord may have clinical consequence, but it will not significantly impact the GPR. Further tools like DVH analysis or clinical goals of treatment targets and OARs could help to better judge the clinical significance of the IDC results.^42^ Detailed commissioning of the IDC tool is essential to understand potential limitations of the IDC system before clinical implementation. In addition, a comprehensive QA program for the beam delivery system must ensure the quality and stability of the beam delivery system on a daily basis. Periodic QA procedures allow to track the stability of the beam delivery via trendline analysis with respect to beam ranges, lateral spot positions and FWHM, homogeneity of 2D fields, dose output in monoenergetic square fields. In addition, we introduced a QA task where five 3D dose distributions are repeatedly measured using our experimental PSQA setup on a monthly basis.

While several homemade and commercial IDC systems are available, the use of log‐file based PSQA is still scarcer in the clinics and the reason for that is intuitively clear: while any clinic is in principle able to export DICOM treatment files from a TPS to run an IDC, log‐files are not necessarily made accessible by all vendors. One key advantage of log‐file based QA is to include the beam delivery and therefore the possibility to check the file transfer and beam deliverability. However, this requires beam time prior the first treatment fraction. As mentioned before, log‐file based IDC was shown to be more effective in detecting data transfer failures.^14^ One could still ask the question about whether file transfer checks really require irradiation, as in principle, the software functionalities should be verified during the system's acceptance and file conversions could be checked by other means. An interesting PSQA proposal independently verifying the accuracy of the calculated physical dose via IDC and the delivered proton fluence (via log‐files) was proposed by Johnson et al.^13^ Due to the dependence between the log‐files and the beam delivery, an additional QA program for the beam monitors was added. Reliable information from the logs is usually limited to beam position and monitor units, while other key parameters like the beam energy are not available at all and must be checked by other means.

In this scope, we are considering to include treatment delivery log‐file analysis in addition to our QA program. Beyond PSQA, the implementation of IDC and log‐file analysis are also key ingredients to facilitate adaptive workflows.

## 5 CONCLUSION

In this work, we presented the commissioning and clinical implementation of an independent dose calculation system for scanned proton beam delivery. The implementation of IDC required an extensive commissioning work. The dosimetric capabilities of the IDC algorithm were tested in 1D/2D/3D. CT calibration validation was verified against measurements. The dose evaluation and analysis tools provided by the IDC system have been commissioned and cross‐checked against other certified medical software. The comprehensive dosimetric commissioning and CT calibration validation were found to be within requirements for clinical usage. Subsequently, the clinical commissioning allowed the determination of clinical gamma pass rate tolerances and fail levels. The beam delivery‐QA program must be comprehensive to ensure treatment safety while reducing experimental PSQA. Fulfilling these essential requirements allowed us to guaranty a safe reduction of experimental PSQA by substitution with IDC and to increase the overall level of QA for our patients. It further enables a more efficient usage of the beam time and to release the medical physics experts from the experimental PSQA burden for scanned proton beam delivery. In addition, the implementation of IDC was found increase flexibility in treatment adaptation.

## AUTHOR CONTRIBUTIONS

Conception and design: Ralf Dreindl, Loïc Grevillot. Data collection: Ralf Dreindl, Marta Bolsa‐Ferruz, Rosa Fayos‐Sola, Fatima Padilla Cabal, Lukas Scheuchenpflug, Alessio Elia, Antonio Amico. Data analysis and interpretation: Ralf Dreindl, Marta Bolsa‐Ferruz, Rosa Fayos‐Sola, Fatima Padilla Cabal, Lukas Scheuchenpflug, Alessio Elia, Antonio Amico, Loïc Grevillot, Antonio Carlino. Manuscript writing: Ralf Dreindl, Loïc Grevillot. Final approval of manuscript: Markus Stock, Loïc Grevillot.

## CONFLICT OF INTEREST STATEMENT

All authors declare that they have no conflicts of interest.
